# Therapeutic outcomes of ranibizumab for zone ii stage 2 retinopathy of prematurity with plus disease

**DOI:** 10.1007/s10384-025-01199-y

**Published:** 2025-05-16

**Authors:** Yoshihiro Nakagawa, Yoshifumi Murayama, Yasuyuki Suzuki, Atsushi Uchiyama, Takahiro Suzuki

**Affiliations:** 1 Department of Ophthalmology, Tokai University School of Medicine, Shimokasuya 143, Isehara, Kanagawa 259-1193 Japan; 2 Department of Pediatrics, Tokai University School of Medicine, Kanagawa, Japan

**Keywords:** Retinopathy of prematurity, Ranibizumab, Reactivation, Persistent avascular retina, Birth weight

## Abstract

**Purpose:**

This study aimed to evaluate the efficacy of intravitreal injections of ranibizumab (IVR) in treating zone II stage 2 retinopathy of prematurity with plus disease (ROP II2+) in preterm infants, focusing on two primary outcomes: reactivation and persistent avascular retina (PAR).

**Study design:**

Retrospective and consecutive case series.

**Methods:**

This retrospective study reviewed the medical records of preterm infants treated with IVR at Tokai University hospital between December 2019 and September 2023. Data on reactivation, PAR, and other clinical outcomes were analyzed using generalized estimating equations to account for correlations between eyes.

**Results:**

Thirty eyes from 16 infants received IVR treatment. Following initial IVR all eyes achieved remission for over one year with IVR alone. Forty percent of these eyes required subsequent treatment due to reactivation, with a median reactivation time of 70 days. One year after initial IVR, PAR was observed in 11 out of the 30 eyes. Lower birth weight was associated with a tendency toward reactivation, and it also showed a significant correlation with the development of PAR.

**Conclusions:**

This research supports the effectiveness of IVR for ROP II2+ over a one-year period. The links between lower birth weight and both reactivation and PAR might guide refinements in therapeutic strategies.

## Introduction

Retinopathy of prematurity (ROP), is characterized by aberrant retinal vascular development; it is a major cause of blindness in preterm infants [1–3]. The pathophysiology of ROP has two distinct phases. Initially, retinal vascularization is suppressed by the hyperoxic environment and is followed by a hypoxia-induced increase in levels of vascular endothelial growth factor (VEGF), resulting in abnormal retinal neovascularization [4, 5]. Laser photocoagulation (LPC) is the definitive treatment for preventing disease progression; however, it is associated with myopia and visual field loss [6].

The introduction of anti-VEGF therapies marks a significant shift in the management of ROP [7]. Bevacizumab, the first anti-VEGF agent used in ROP, showed promising results, particularly in zone I disease, by reducing the need for retreatment compared to laser therapy [8]. However, there are concerns on its systemic effects and potential impact on neurodevelopment due to its long half-life, hence the need for alternative agents. Currently, in addition to bevacizumab, ranibizumab, aflibercept, and conbercept are used as anti-VEGF therapies for ROP, broadening the range of treatment options available. With these anti-VEGF medications for ROP, there is a need for caution regarding reactivation, and the presence of peripheral retinal avascular zones, known as persistent avascular retina (PAR), is frequently observed post-treatment.

Ranibizumab, a humanized monoclonal antibody fragment that inhibits VEGF-A, is a potential alternative as it has a shorter half-life, potentially offering a safer profile for preterm infants. Clinical trials, such as the RAINBOW study [9], show that ranibizumab was as effective as laser therapy in regressing ROP but with fewer unfavorable structural outcomes and a lower incidence of high myopia. The RAINBOW study is a landmark study that shows the efficacy of ranibizumab for ROP, with treatment eligibility criteria similar to those for type 1 ROP as defined in the ETROP study [10]. However, zone II stage 2 with plus disease (II2+) was excluded from that study. Furthermore, no reports have elucidated the efficacy of ranibizumab in II2+ condition. This study aimed to examine the therapeutic efficacy of ranibizumab for ROP II2+, focusing on two primary outcomes: reactivation and PAR.

## Subjects and methods

### Subjects

This study was approved by the Institutional Review Board of Tokai University for Clinical Research (Approval number: #24R159-001 H) and conformed to the principles outlined in the Declaration of Helsinki.

We performed a retrospective review of medical records of consecutive infants who received intravitreal injections of ranibizumab (IVR) as initial treatment for ROP II2+ between December 2019 and September 2023. Infants were included if they had been followed for at least 1 year after receiving IVR. Parents of infants with ROP II2+ were informed about both IVR and LPC treatment options, and those who opted for IVR were included in this study. Infants who did not require additional treatment after the initial IVR during the 1-year follow-up period were classified into the no reactivation group, while those who required further intervention were classified into the reactivation group.

### Fundus examination

Pupils of the infants were dilated with 0.5% tropicamide and 0.5% phenylephrine eye drops before examination with a binocular inverted stereoscope. The fundus was observed with a 28D lens to determine ROP progression. The latest ROP classification was defined in 2021 (ICROP3) [11], wherein findings related to reactivation following anti-VEGF therapy are mentioned. Although our study included cases from before 2021, the location was classified according to the ICROP3 definition from the medical record. Cases were classified as treatment-required reactivation whenever they exhibited the self-limiting demarcation line and retinal vascular tortuosity in the posterior pole that tended to worsen over 1 week; additional treatment for these cases was planned accordingly. Persistent avascular retina (PAR) after IVR was also defined as an extended avascular area on the temporal side of the retina by more than two optic disc diameters from the orra serrata.

### Treatment

Within 3 days of ROP II2+ diagnosis, IVR was administered with the assistance of a neonatologist. Infants were administered a single intravenous dose of pentazocine (0.5 mg/kg) for sedation and atropine (0.01 mg/kg) to prevent bradycardia, after which they were wrapped in a towel for immobilization. Next, 0.4% oxybuprocaine ophthalmic anesthesia was administered, the eyelid was disinfected with povidone iodine, the conjunctival sac was washed, an eyelid speculum was applied. Following this, ranibizumab 0.2 mg/0.02 mL was injected into the vitreous cavity through the sclera using a 34-gauge needle 1.0–1.5 mm away from the limbus. To prevent infection, gatifloxacin eye drops were administered four times daily for 1 week.

### Follow-up

Complications and effects of treatment were evaluated using a binocular inverted microscope and a portable slit lamp 1, 3, 4 days, and 1 week after IVR. Thereafter, the eyes were examined every 1 or 2 weeks according to the condition of the retina. When additional IVR was performed, the interval between examinations was shortened similarly. However, when ROP remission for >3 months was achieved, the interval was extended to >1 month.

### Data collection

Ophthalmological data were extracted from medical records and included the week of gestation at the time of treatment, age, presence of reactivation, time to reactivation, and presence of PAR. Data on birth status (sex, gestational age [GA], and birth weight [BW]) and other systemic information such as oxygen administration period, respiratory distress syndrome, intraventricular hemorrhage and blood transfusion were also obtained.

### Statistical analysis

Statistical analyses were conducted using StatFlex version 7 (Artech Co.) and SPSS Statistics version 26.0 (IBM Company). Analysis including all treated eyes used the Mann-Whitney U test or McNemar test. Because both bilateral and unilateral treatment are included, for investigating the factors related with reactivation and PAR, we used generalized estimating equations (GEE) model to account for inter-eye correlation in a subject. In the GEE model, using unstructured correlation structure, the occurrence of ROP reactivation was a dependent variable, and eye side and background factors of patients were independent variables.* P* values <0.05 were considered significant.

## Results

Overall, 30 consecutive eyes from 16 infants with ROP II2+ received initial IVR. The general birth characteristics and IVR details are shown in Table 1. In cases involving both eyes, the data are treated as duplicates; the median GA and its interquartile range (IQR) of the infants was 25.8 (24.9–26.4) weeks, and the median BW was 682.0 (IQR 564.5–743.2) g. The median postmenstrual age (PMA) at initial IVR was 35.4 (IQR 34.6–39.0) weeks. All cases of ROP were classified within zone II; posterior zone II was identified in eight eyes (26.7%). Following the initial IVR, treatment-required reactivation of ROP occurred in 12 eyes (40.0%), with a median interval between the first and second IVR of 70.0 (IQR 62.0–89.0) days. Among the eyes that required a second IVR, only one eye underwent a third IVR due to repeated ROP reactivation.

**Table 1:** Demographic Characteristics of Eyes Treated with IVR

Total, eyes	30	*
bilateral/unilateral, cases	14/2	
Boys/girls, cases	12/18	*
GA, median (IQR), weeks	25.8 (24.9–26.4)	*
BW, median (IQR), g	682.0 (564.5–743.2)	*
Posterior zone II, eyes (%)	8 (26.7)	*
PMA at initial IVR, median (IQR), weeks	35.4 (34.6–39.0)	*
Second IVR for reactivation, eyes (%)	12 (40.0)	*
Duration from initial injection, median (IQR), days	70.0 (62.0–89.0)	*
Third IVR for reactivation, eyes (%)	1 (3.3)	

*IVR* Intravitreal injection of ranibizumab, *GA* Gestational age, *BW* Birth weight, *IQA* Interquartile range, *PMA* Postmenstrual age

^*^In cases involving both eyes, the data are treated as duplicates.

Table 2 shows the detailed comparison of clinical courses of IVR between the reactivation and no reactivation groups along with the results from the statistical analyses, treating data as duplicates in cases involving both eyes. Posterior zone II at the time of initial IVR was significantly more common in the reactivation group (P=0.018), while PMA and PNA were similar in both groups (P=0.176 and 0.983 respectively). No patients in either group required further LPC or surgical interventions other than IVR. Additionally, there were no occurrences of complications such as cataracts or endophthalmitis due to IVR. One year after the initial IVR, the incidence of PAR was significantly higher in the reactivation group, where it was observed in all cases, compared to the no reactivation group (P=0.004).

**Table 2:** Clinical Course of IVR in reactivation and no reactivation group

	Reactivation (n=12)	No reactivation (n=18)	*P* values	
Posterior zone II (%)	6 (50.0)	2 (10.0)	0.018^†^	*
PMA at initial IVR, median (IQR), weeks	35.2 (34.5–36.3)	36.1 (34.5–38.7)	0.176	*
PNA at initial IVR, median (IQR), days	70.0 (68.0–77.0)	70.5 (61.0–89.8)	0.983	*
Other treatments besides IVR (%)	0 (0)	0 (0)		
PAR (%)	12 (100.0)	9 (50.0)	0.004^‡^	*

*IVR* Intravitreal injection of ranibizumab, *PMA* Postmenstrual age, *IQA* Interquartile range, *PNA* Postnatal age

^*^In cases involving both eyes, the data are treated as duplicates.

^†^*P*<0.05, Mann-Whitney U test

^‡^*P*<0.05, McNemar test

The characteristics of patients in the reactivation and no reactivation groups of ROP after the initial IVR are shown Table 3. Six infants experienced a reactivation after the first IVR, while ten did not. From the estimates of GEE regarding the background factors in this table, lower BW had odds ratio 0.993 (95%CI 0.986–1.000), and this indicates a negative association tendency between ROP reactivation with P-value of 0.057, though no significant differences or trends were observed in the other factors.

**Table 3:** Comparison the presence or absence of reactivation based on patient characteristics

Background	Reactivation (n=6)	No reactivation (n=10)
Gender (boy/girl)	3/3	3/7
GA, median (IQR), weeks	25.2 (24.4–25.8)	26.1 (25.8–27.1)
BW, median (IQR), g	634.5 (436.0–682.0)	736.0 (670.0–905.0)
Oxygen administration period, median (IQR), days	52.5 (35.0–70.0)	27.0 (18.0–46.0)
Respiratory distress syndrome (%)	4 (66.7)	9 (90.0)
Patent ductus arteriosus (%)	4 (66.7)	7 (70.0)
Intraventricular hemorrhage (%)	1 (16.7)	1 (10.0)
Blood transfusion (%)	5 (83.3)	7 (70.0)

*GA* Gestational age, *IQA* Interquartile range, *BW* Birth weight

Table 4 shows the presence or absence of PAR as an outcome one year after the initial IVR, along with the same background factors as in Table 3. Eleven patients were found to have findings of PAR, while 5 did not have such findings. From the estimates of GEE, lower BW had odds ratio 0.992 (95%CI 0.985–0.999), and this shows the significant association between PAR with a P-value of 0.019.

**Table 4:** Comparison the presence or absence of PAR based on patient characteristics

Background	PAR (n=11)	No PAR (n=5)
Gender (boy/girl)	5/6	2/3
GA, median (IQR), weeks	25.5 (24.6–25.8)	26.5 (26.4–27.1)
BW, median (IQR), g	667.0 (494.0–708.5)	746.0 (726.0–905.0)
Oxygen administration period, median (IQR), days	46.0 (32.0–63.0)	23.0 (18.0–25.0)
Respiratory distress syndrome (%)	9 (81.8)	4 (80.0)
Patent ductus arteriosus (%)	6 (54.5)	3 (60.0)
Intraventricular hemorrhage (%)	1 (9.0)	1 (20.0)
Blood transfusion (%)	9 (81.8)	3 (60.0)

*PAR* Persistent avascular retina, *GA* Gestational age, *IQA* Interquartile range, *BW* Birth weight

## Discussion

This study evaluated the efficacy of IVR on ROP II2+ with two primary outcomes, reactivation and PAR. We also assessed the associated risk factors for reactivation or PAR. Of the 30 eyes treated with IVR, 40% eyes required additional treatment due to reactivation. Nevertheless, all eyes achieved up to one year remission through IVR monotherapy, and lower BW tended to be associated with ROP reactivation and was a significant associated factor for PAR.

Preventing retinal detachment is a critical objective in ROP management. LPC is the primary treatment approach, but recent advances in anti-VEGF therapies provide alternative treatment options [12]. These therapies include bevacizumab, ranibizumab, aflibercept, and conbercept, all of which have demonstrated efficacy in halting disease progression by suppressing abnormal retinal neovascularization. Although IVR offers significant initial disease regression, it is commonly associated with a higher reactivation rate compared to LPC [13, 14]. Zhang et al. show that 44% of zone II ROP cases treated with IVR experienced reactivation, compared to just 4% in those treated with LPC [15]. There are reports on reactivation following IVR for zone II ROP, but none have evaluated the differences between stages 2 and 3 [16–18]. In contrast, Xia et al. provide partial data regarding ROP II2+, and found that 49.0% of II2+ cases showed reactivation [19]. While this outcome differs from our data by nine points, it is difficult to make a direct comparison due to differences in the definition of reactivation, as well as variations in patients' birth and postnatal backgrounds between the two studies. Nevertheless, the data reaffirm that reactivation after IVR is not uncommon.

The median duration from the initial IVR to the second IVR due to reactivation in this study was 70.0 days, longer than the reactivation time of 53.3 and 58.1 days reported by previous studies [20, 21]. Our findings may be explained by the time needed to recognize treatment-required reactivation, inform parents, and schedule treatment. Thus, the interval to reactivation in this study is likely comparable to previous results. Although remission was maintained for one year after initial IVR treatment, long-term monitoring is essential due to potential complications such as exudative retinal detachment and relapses occurring up to six years post-treatment [22, 23].

Several factors have been proposed as predictors of reactivation following anti VEGF therapy for ROP, including preretinal hemorrhage, anemia, sepsis, BW, and PMA at first treatment [21, 24–26]. In this study, GA was similar between the reactivation and no reactivation groups, but median BW was apparently lower in the no reactivation group, and GEE analysis showed a tendency of being associated with reactivation. As for the limitation of sample size, further studies are needed to clarify the association between BW and ROP reactivation. If conditions at birth like BW are linked to reactivation after IVR, it might allow for easier screening of reactivation risk in planning the treatment ROP II2+.

In our study, PAR, the incomplete vascularization of the peripheral retina that can lead to retinal detachment and other complications, was frequently observed. Wu et al. show that PAR rates were similar between patients treated with IVR and those with zone II ROP who experienced spontaneous remission, indicating that incomplete vascularization may be part of the natural disease course [27]. On the other hand, there is research that shows that PAR in children aged 4 to 8 years is significantly more common in cases of ROP that have received anti-VEGF therapy than in cases with ROP that has not developed or has spontaneously regressed [28]. While the long-term effects of PAR remain unknown, fluorescein angiography is beneficial for a detailed assessment of vascular abnormalities [27, 29]. In our study, PAR was observed in a relatively short period, 1 year after the initial IVR for ROP II2+, so future studies are needed to clarify the long-term implications of PAR.

In this study, all eyes were able to maintain remission for over one year with IVR as monotherapy. While the high reactivation rates and the presence of PAR are considered drawbacks of IVR compared to LPC, one key advantage of IVR is the relatively minor refractive errors observed after treatment, a finding supported by several meta-analyses [30, 31]. From this perspective, although the course of refraction was not followed in this study, IVR monotherapy offers notable benefits. However, Tsai et al. caution against its use for zone II ROP due to a higher rate of reactivation [7]. They recommend LPC following IVR reactivation to mitigate systemic risks associated with VEGF suppression and address retinal ischemic stress, despite Marlow et al. showing no systemic adverse effects five years post-IVR [32]. In terms of refractive errors after LPC, Mori et al. found that the severity of myopia increases with the number of laser spots used [33]. Notably, in cases of ROP II2+ reactivation, fewer laser spots are needed compared to zone I or aggressive ROP. Therefore, combining IVR and LPC in ROP II2+ cases may limit systemic drug exposure and reduce the likelihood of significant refractive errors.

This study has several limitations. It is a single-center retrospective study with a small sample size, some of which included bilateral cases. Additionally, outcomes beyond 1 year were not evaluated, and factors closely associated with systemic conditions post-IVR may have been missed.

In conclusion, we showed that IVR is effective for short-term treatment of ROP II2+. In all eyes, remission of ROP was maintained for more than one year with IVR alone. Among the severities of ROP requiring treatment, II2+ is relatively mild, but the reactivation rate was 40%. PAR was particularly common in the reactivation group, underscoring the importance of future monitoring. The reactivation and PAR are suggested to be associated with BW.
